# ARID1B Immunohistochemistry Is an Important Test for the Diagnosis of Dedifferentiated and Undifferentiated Gynecologic Malignancies

**DOI:** 10.3390/cancers15174229

**Published:** 2023-08-24

**Authors:** Basile Tessier-Cloutier

**Affiliations:** 1 Department of Pathology, McGill University, Montreal, QC H3A 2B4, Canada; basile.tessiercloutier@mcgill.ca; Tel.: +1-(514)-934-1934; 2 Division of Pathology, McGill University Health Centre, Montreal, QC H4A 3J1, Canada; 3 Cancer Research Program, Research Institute of the McGill University Health Centre, Montreal, QC H4A 3J1, Canada

**Keywords:** ARID1B, SWI/SNF, endometrial cancer, ovarian cancer, immunohistochemistry, dedifferentiated carcinoma, undifferentiated carcinoma, SMARCA4, SMARCB1

## Abstract

**Simple Summary:**

Mounting evidence show that dedifferentiated and undifferentiated endometrial and ovarian carcinomas (DDC/UDC) are clinically distinct entities with rapid progression, poor response to adjuvant therapy, and grim outcome, even when compared to other high-grade gynecologic malignancies. Unfortunately, they are very challenging to diagnose and often receive a non-specific or ambiguous diagnosis. Immunohistochemical tests for core SWI/SNF proteins (i.e. ARID1B, SMARCA4, and SMARCB1) offer high specificity for DDC/UDC, low cost, and fast turnaround time, unlike DNA sequencing-based assays which currently have very limited use in predicting inactivation core SWI/SNF subunits. Recent reports show that among the known core SWI/SNF proteins, ARID1B inactivation are most common in these tumors, yet this test is rarely available even in tertiary centers. In this opinion I stress the importance of including ARID1B along with other core SWI/SNF proteins in the diagnostic workup of DDC/UDC.

**Abstract:**

Dedifferentiated and undifferentiated endometrial and ovarian carcinomas (DDC/UDC) are aggressive malignancies defined by morphologic and molecular undifferentiation, and associated with core SWI/SNF deficiency. Their main differential diagnoses include high-grade endometrial and ovarian carcinomas that often show overlapping morphologic and molecular profiles. Loss of cell lineage markers expression by immunohistochemistry (IHC) is commonly used to assist diagnosis, but it has poor specificity, while core SWI/SNF deficiency is much more specific. Approximately half of SWI/SNF-deficient DDC/UDC are associated with loss of ARID1B expression, yet, unlike the other core SWI/SNF proteins (SMARCA4 and SMARCB1), this test is rarely available, even in tertiary centers. Mutational testing for ARID1B is increasingly common among targeted DNA sequencing panels, but it is difficult to interpret in the absence of IHC results. Overall, the importance of including ARID1B IHC as part of the routine panel for undifferentiated gynecologic malignancies should be emphasized, especially as SWI/SNF inactivation is becoming a necessary biomarker for diagnostics, clinical management, and clinical trial enrollment.

## 1. Discussion

### 1.1. Background

Switch/sucrose non-fermentable (SWI/SNF) is a protein complex with an important role in cell development. It allows the selective expression of certain genes within different tissue types through the manipulation of chromatin organization and by counterbalancing another epigenic modulator known as the polycomb repressive complex [1,2]. Inactivation of the SWI/SNF complex through alterations of core subunits (*SMARCA4*, *SMARCB1*, and *ARID1B*) has been strongly associated with dedifferentiated and undifferentiated endometrial and ovarian carcinomas (DDC/UDC) but is also reported in dedifferentiated and undifferentiated malignancies from many different tissues, including gastrointestinal, hepatobiliary, genitourinary, head and neck, thoracic, mammary, and neurologic [3,4,5,6,7,8,9,10]. DDC/UDC are very aggressive malignancies often presenting at high stage and with median overall survival under one year, yet they are still underrecognized [11,12]. In the endometrium, there is a wide range of reported incidence rates (1–9% of endometrial carcinomas), likely in part a reflection of their subjective diagnostic criteria that contribute to the misinterpretation of these entities [13,14,15]. In the ovary, DDC/UDC are less common, but their true incidence is unknown. Essential diagnostic features include a combination of morphologic (e.g., sheet-like growth without glandular or papillary architecture) and differentiation marker expression (e.g., loss or reduced expression of CK7, PAX8, ER, and claudin-4). Loss of expression of a core SWI/SNF protein can help confirm the diagnosis, but it is not an essential feature. When an undifferentiated carcinoma is associated with a “differentiated” carcinoma (usually a low-grade endometrioid carcinoma), the lesion is referred to as dedifferentiated carcinoma (DDC); however, when no differentiated component is identified, despite adequate sampling, it is referred to as undifferentiated carcinoma (UDC). Inactivation of the SWI/SNF complex in these tumors is believed to drive the phenotypic undifferentiation and oncogenic behavior through epigenetic alterations that create an imbalance between self-renewal and proliferation gene expression [1]. Morphologically, this shift is seen as a transition into a primitive undifferentiated appearance including cellular discohesion and monomorphism, as well as a shapeless architecture. This is accompanied by decreased expression of cell lineage markers [16]. Loss of SMARCA4, SMARCB1, and ARID1B protein expression by immunohistochemistry (IHC) has been shown to be an effective way to predict mutation in their associated genes [4]. Other SWI/SNF alterations in non-core subunits (e.g., ARID1A) are commonly reported but are not well associated with the undifferentiated morphology and highly aggressive clinical behavior seen in core SWI/SNF-deficient undifferentiated tumors [12,17].

### 1.2. The Co-Loss of ARID1A and ARID1B Expression Is Associated with an Undifferentiated Phenotype

ARID1A and ARID1B are alternate but necessary DNA-binding subunits of the SWI/SNF complex (BRM-associated factors configuration) [18,19]. Isolated alterations of the *ARID1A* gene are common in many differentiated epithelial carcinomas, especially in the gynecologic tract, where they are associated with clear cell carcinoma but also endometrioid and mucinous carcinoma [20,21,22]. Likely because of the redundant role of ARID1B, ARID1A inactivation alone usually does not appear to be sufficient to drive undifferentiation. The inactivation of both genes, on the other hand, has been shown to trigger undifferentiation in DDC/UDC in the gynecologic tract as well as many other sites [3,17]. ARID1B-deficient DDC/UDC, like those with other core SWI/SNF deficiencies (i.e., SMARCA4 and SMARCB1), are discohesive, monomorphic, and mitotically active tumors growing in sheets; they lose or have reduced expression of markers of cell of origin and are commonly associated with mismatch repair (MMR) deficiency. Interestingly, isolated ARID1B loss is not well reported, and it is unclear if it can drive undifferentiation in an *ARID1A* wild-type malignancy. Loss of ARID1B expression has been shown to be a specific biomarker for the diagnosis of DDC/UDC, even without knowledge of the ARID1A status [23]. This is akin to small cell carcinoma of the ovary hypercalcemic type (SCCOHT), where the overwhelming majority of cases show co-loss of SMARCA4 and SMARCA2, yet SMARCA4 IHC alone is regarded as sufficient to confirm diagnosis [24]. This predilection for a certain core SWI/SNF subunit in different tumor types is still poorly understood but may reflect the plasticity in the configuration of the different SWI/SNF analogs, depending on the tumor’s tissue of origin and cellular state. The mechanisms behind this contextual configuration of the SWI/SNF complex are being actively researched but go beyond the scope of this commentary.

### 1.3. ARID1B IHC Should Be Included as Part of the Diagnostic Panel for Undifferentiation in Gynecologic Malignancies

Series of DDC/UDC show that roughly 50% have loss of expression for SMARCA4, SMARCB1, or ARID1B [12,17,25]. It is still unclear if the other half should be regarded as DDC/UDC or poorly differentiated carcinoma. Early evidence suggests that inactivation of the SWI/SNF complex can identify the more aggressive tumors among those with undifferentiated morphology; however, more research is needed to validate this finding [12]. Nevertheless, with the increasing number of clinical trials targeting SWI/SNF complex inactivation, there is a need to identify lesions with loss of the core SWI/SNF subunits. In the endometrium, about half of the DDC/UDC with aberrant core SWI/SNF expression show loss of ARID1B, while the other half lose SMARCA4 expression. Loss of SMARCB1 is also reported in DDC/UDC, but it is much less frequent [12,17,25]. This distribution is mirrored in ovarian DDC/UDC, where about half show loss of ARID1B IHC [11]. So far, there have not been noticeable clinical or morphologic differences associated with ARID1B that differentiate it from the other two core subunits in DDC/UDC, and all are currently classified under the same umbrella terminology of dedifferentiated or undifferentiated carcinomas. In the gynecologic tract, just like SMARCA4 and SMARCB1, ARID1B loss is almost exclusively seen in undifferentiated malignancies [23]. However, ARID1B loss has not yet been reported in pediatric and young adult undifferentiated malignancies, such as SCCOHT and SMARCA4-deficient uterine sarcoma (SDUS), or malignant rhabdoid tumors of the kidney (MRT), and atypical teratoid rhabdoid tumor (ATRT). Since their morphologic features overlap greatly, this distinction can be very helpful in some cases to support the diagnosis of DDC/UDC. Unlike these other undifferentiated malignancies, DDC/UDC, including those with ARID1B deficiency, usually occur in older women (median ~60 years old) and are associated with significant mutational burden [12,26,27]. Their mutational profile includes well-known endometrial cancer-associated genes [27]. More recently, methylation profiling has also shown that UDC, with ARID1B or SMARCA4 deficiency, are separate from SCCOHT, SDUS, and MRT [28].

In many centers, the workup of dedifferentiated and undifferentiated gynecologic malignancies typically includes IHC tests for SMARCA4 and SMARCB1. These markers have now been used for many years in assisting the diagnoses of other undifferentiated malignancies, usually in children and young adults, involving sites such as the central nervous system (ATRT and poorly differentiated chordoma), kidneys (MRT), ovaries (SCCOHT), and soft tissue (epithelioid sarcoma) [7,29,30,31]. As such, they are commonly available in many Western pathology laboratories, and most pathologists are comfortable with their interpretation. The ARID1B IHC, on the other hand, is not available in the clinical setting, except for a handful of institutions in the world. Current literature, as discussed in the previous paragraph, certainly calls for this to change, and hopefully the ARID1B becomes a standard diagnostic tool for undifferentiated malignancies in the gynecologic tract and beyond. Although loss of core SWI/SNF protein expression is not yet a required criteria for the diagnosis of DDC/UDC in the World Health Organization (WHO) Classification of Tumours, adding ARID1B to SMARCA4 and SMARCB1 is very helpful in the diagnosis of a significant number of cases; cases for which the pathologist may have otherwise had to use a nonspecific diagnostic terminology [32]. Overall, given its prevalence and role for prognostication and clinical trial selection, ARID1B is an important biomarker as part of the diagnostic workup of DDC/UDC.

### 1.4. ARID1B IHC May Be a More Efficient Biomarker of SWI/SNF Inactivation Than ARID1B Mutational Profiling

With the emergence of large targeted DNA sequencing panels, the mutational profiling of *ARID1B* is becoming increasingly common in tertiary institutions; however, the interpretation of these results remains a challenge. Since *ARID1B* inactivation typically requires two pathogenic mutations positioned in trans or one mutation associated with loss of heterozygosity (LOH), it is difficult to confirm using traditional next-generation sequencing approaches [11]. Some bioinformatic techniques, such as FACET, can help identify LOH in some cases, but confirming complete loss of function is not possible in most cases [33]. This is further complicated in hypermutated cases with MMR deficiency, where non-inactivating mutations in the *ARID1B* gene are common and usually associated with a typical low-grade endometrioid morphology. The IHC approach benefits from having a faster turnaround time, easier interpretation, and a cheaper cost. There are well-known flaws of using IHC to detect mutations, such as a non-functional protein causing false-positive expression or false-negative expression in suboptimal tissue fixation, but they have not been well defined for the ARID1B IHC. The ARID1B 2D2 Abnova clone, which has been used for most publications involving DDC/UDC, has been shown to be relatively robust. In some cases, ARID1A IHC has been used to help with the interpretation of borderline cases, similarly to how SMARCA2 IHC can be used to help with the interpretation of SMARCA4. Because the loss of expression of ARID1B is almost exclusively seen in tumors with synchronous inactivation of the *ARID1A* gene, the retention of ARID1A expression supports that the ARID1B subunit is also intact. In difficult cases, a DNA sequencing assay including the mutational status of *ARID1A* and *ARID1B* genes may be helpful to determine SWI/SNF status in combination with the ARID1B IHC.

### 1.5. ARID1B-Deficient Tumors Show Very Distinct Methylation Profiles Compared to Those with Intact SWI/SNF Expression

Prior studies have shown that methylation profiling can be a reliable tool to assess tumor subtypes [34]. Using the same assay in undifferentiated endometrial carcinoma with loss of ARID1B expression shows that they have a different signature to endometrial carcinomas with intact core SWI/SNF status [28]. Instead, their profile overlaps with SMARCA4-deficient undifferentiated endometrial carcinoma. This likely reflects the deep epigenetic shift that results from the inactivation of the SWI/SNF complex in these tumors. More importantly, it highlights that DDC/UDC with the inactivation of a core SWI/SNF complex are molecularly distinct to DDC/UDC and other tumors with intact core SWI/SNF expression. It is still unclear if the type of SWI/SNF inactivation, ARID1B, SMARCA4, or SMARCB1, have distinctive effects on the methylation profile, but certainly the molecular context, such as inactivating MMR mutations and global methylation status, can be different from one DDC/UDC to another.

These findings, along with evidence showing significantly worse outcomes in DDC/UDC with core SWI/SNF inactivation, support that these entities should be classified separately to other morphologically undifferentiated endometrial and ovarian tumors. The “SWI/SNF-deficient undifferentiated malignant neoplasm” or SDUMN nomenclature has been suggested as a way to reflect the distinct morphologic and molecular features of this disease. The current diagnostic criteria for DDC/UDC are very inclusive and as such likely create a heterogenous groups of tumors. However, before achieving consensus on a new terminology, more work is needed to validate the current literature.

### 1.6. Conclusions

DDC/UDC are becoming increasingly recognized as a distinct and extremely aggressive malignancy with poor response to conventional therapies. The accurate diagnosis and characterization of the SWI/SNF status will be essential to further our understanding of these lesions and offer appropriate clinical trials to our patients. It is also this meticulous clinical and molecular characterization that will clarify the need for a dedicated terminology.

SMARCA4 and SMARCB1 IHC alone miss a significant number of DDC/UDC with SWI/SNF inactivation, while the mutational profiling of *ARID1B* with DNA sequencing is often inconclusive. When properly optimized, ARID1B IHC offers a robust, fast, and cheap test to assess SWI/SNF status, and it has been shown to be very helpful for the diagnosis of DDC/UDC. Its application will likely also be useful for the characterization and diagnosis of non-gynecologic undifferentiated malignancies. Going forward, there is an obvious need to better characterize the interplay between the different SWI/SNF mutations and their resulting effects on protein expression, morphology, and clinical behavior.

## Data Availability

No new data presented.
